# Employing Molecular Phylodynamic Methods to Identify and Forecast HIV Transmission Clusters in Public Health Settings: A Qualitative Study

**DOI:** 10.3390/v12090921

**Published:** 2020-08-22

**Authors:** Shannan N. Rich, Veronica L. Richards, Carla N. Mavian, William M. Switzer, Brittany Rife Magalis, Karalee Poschman, Shana Geary, Steven E. Broadway, Spencer B. Bennett, Jason Blanton, Thomas Leitner, J. Lucas Boatwright, Nichole E. Stetten, Robert L. Cook, Emma C. Spencer, Marco Salemi, Mattia Prosperi

**Affiliations:** 1 Department of Epidemiology, College of Public Health and Health Professions & College of Medicine, University of Florida, Gainesville, FL 32610, USA; vrichh@ufl.edu (V.L.R.); n.e.stetten@phhp.ufl.edu (N.E.S.); cookrl@ufl.edu (R.L.C.); m.prosperi@ufl.edu (M.P.); 2 Emerging Pathogens Institute, University of Florida, Gainesville, FL 32610, USA; cmavian@ufl.edu (C.N.M.); brittany.rife@epi.ufl.edu (B.R.M.); msalemi@ufl.edu (M.S.); 3 Department of Pathology, Immunology, and Laboratory Medicine, College of Medicine, University of Florida, Gainesville, FL 32610, USA; 4 Division of HIV/AIDS Prevention, Centers for Disease Control and Prevention, Atlanta, GA 30322, USA; bis3@cdc.gov (W.M.S.); Karalee.Poschman@flhealth.gov (K.P.); 5 Florida Department of Health, Division of Disease Control and Health Protection, Bureau of Communicable Diseases, HIV/AIDS Section, Tallahassee, FL 32399, USA; Steven.Broadway@flhealth.gov (S.E.B.); Emma.Spencer@flhealth.gov (E.C.S.); 6 Division of Public Health, Injury and Violence Prevention Branch, North Carolina Department of Health and Human Services, Raleigh, NC 27699, USA; shana.geary@dhhs.nc.gov; 7 Florida Department of Health, Bureau of Public Health Laboratories, Jacksonville, FL 32202, USA; Berry.Bennett@flhealth.gov (S.B.B.); Jason.Blanton@flhealth.gov (J.B.); 8 Theoretical Biology & Biophysics Group, Los Alamos National Laboratory, Los Alamos, NM 87545, USA; tkl@lanl.gov; 9 Department of Plant and Environmental Sciences, Clemson University, Clemson, SC 29634, USA; jboatw2@clemson.edu; 10 Advanced Plant Technology Program, Clemson University, Clemson, SC 29634, USA

**Keywords:** molecular epidemiology, HIV phylogenetics, surveillance, HIV prevention, qualitative research, focus groups

## Abstract

Molecular HIV surveillance is a promising public health strategy for curbing the HIV epidemic. Clustering technologies used by health departments to date are limited in their ability to infer/forecast cluster growth trajectories. Resolution of the spatiotemporal dynamics of clusters, through phylodynamic and phylogeographic modelling, is one potential strategy to develop a forecasting tool; however, the projected utility of this approach needs assessment. Prior to incorporating novel phylodynamic-based molecular surveillance tools, we sought to identify possible issues related to their feasibility, acceptability, interpretation, and utility. Qualitative data were collected via focus groups among field experts (*n* = 17, 52.9% female) using semi-structured, open-ended questions. Data were coded using an iterative process, first through the development of provisional themes and subthemes, followed by independent line-by-line coding by two coders. Most participants routinely used molecular methods for HIV surveillance. All agreed that linking molecular sequences to epidemiological data is important for improving HIV surveillance. We found that, in addition to methodological challenges, a variety of implementation barriers are expected in relation to the uptake of phylodynamic methods for HIV surveillance. The participants identified several opportunities to enhance current methods, as well as increase the usability and utility of promising works-in-progress.

## 1. Introduction

Molecular HIV surveillance methods, including phylogenetics and phylodynamics, are considered a promising approach to better detect and respond to emerging HIV transmission clusters [1]. In fact, this is a key component of the United States (US) Department of Health and Human Services’ Ending the HIV Epidemic: A Plan for America (EHE) in the US, which seeks to reduce new HIV infections by 90% by 2030 [2]. Molecular surveillance methods are enabled through the collection of HIV genetic sequences from people living with HIV (PLWH), as a part of routine clinical care to monitor HIV drug resistance. Phylogenetic analysis of HIV sequences permits the detection of molecular transmission clusters, which provide insight into the underlying transmission network of PLWH whose disease may or may not be diagnosed or who may not be in care [3].

Many of the molecular surveillance methods employed by health departments across the US rely on viral genetic distances between sequences to define HIV transmission clusters. These methods include the HIV-TRAnsmission Cluster Engine (HIV-TRACE or Secure-HIV-TRACE) [4], PhyloPart [5], ClusterPicker [6], and MicrobeTrace (https://github.com/CDCgov/MicrobeTrace/wiki, accessed on 6 May 2020). Although these approaches provide a valuable cross-sectional snapshot of molecularly-linked individuals, including summaries about the demographic, geographic, and temporal characteristics of PLWH within clusters, they are often retrospective in nature and lack the ability to monitor or forecast cluster dynamics in real time. The ability to accurately predict which clusters are likely to grow (versus stabilize or shrink) over time is critical to inform targeted public health interventions aimed at curbing transmission. To that end, incorporation of spatiotemporal information in molecular modelling, via phylodynamic and phylogeographic frameworks, has been proposed as one potential strategy to help resolve cluster dynamics and forecast future trajectories [7]; however, the development of molecular-based forecasting methods is at a nascent stage, and their projected utility in public health settings needs assessment. 

Lessons from HIV implementation research have underscored the importance of human-centered design approaches when implementing evidence-based tools for public health [8]. This approach involves engaging end-users as co-designers to better appreciate their priorities and identify actionable insights to more effectively deploy new technologies that support decision-making. Prior studies have examined the attitudes of stakeholders on the use of HIV molecular epidemiology in public health [9,10,11,12,13]. These studies have explored many of the ethical challenges surrounding the use of HIV molecular data in research and public health, including data privacy, informed consent, and public trust [10,13], concluding that most stakeholders believe the benefits of HIV molecular epidemiology outweigh the risks [12]. To date, however, consideration of the feasibility, utility, and usability of more advanced molecular phylodynamic methods for public health surveillance and intervention is not well represented in the scientific literature. 

To assess the needs and challenges of developing and employing molecular phylodynamic models for HIV surveillance and cluster characterization, we conducted a qualitative study through focus groups using a human-centered design approach. Focus groups offer a flexible and effective way to generate stakeholder input and explore the dimensions of acceptability to ultimately increase the uptake and success of proposed intervention strategies [14]. We gathered experts in molecular epidemiology, infectious disease research and surveillance, and public health officials from the Centers for Disease Control and Prevention (CDC) and the Florida Department of Health (FDOH) based on their academic/professional roles and/or demonstrated histories of prior publications on HIV surveillance or molecular epidemiology topics. We concentrated our study in Florida because the state consistently ranks among the top five states in terms of new HIV diagnoses in the US [15], with seven Florida counties identified as core regions for the 2019 EHE plan [2]. New strategies are therefore needed to prevent HIV transmission in Florida.

## 2. Materials and Methods 

### 2.1. Study Population

A total of three focus group sessions were conducted, each composed of 5–7 participants. Participants were recruited using a combination of purposive, convenience, and snowball sampling, inviting experts in molecular epidemiology, phylogenetics, and public health from the FDOH, academia, other government agencies, and non-profit organizations. We considered experts to be those with a prior publication record on related topics (e.g., HIV surveillance, molecular epidemiology) and/or those in relevant professional/academic positions (e.g., research scientists, public health professionals). In the months leading up to the focus group sessions, the principal investigators and co-investigators compiled a list of potential participants, collecting suggestions from the FDOH and other infectious disease research scientists, and sent out invitations. Participants were given the options to participate in person or remotely and to participate in one or multiple sessions, depending on their availability and area of expertise.

### 2.2. Ethics and Consent

Prior to data collection, we received approval from the University of Florida Institutional Review Board (approval date: 24 September 2018; reference number: IRB201801983). Written informed consent was obtained from all participants. The participants were informed that the objective of the focus group was to gather expert insight to determine how phylodynamic forecasting tools can be effectively implemented and employed by state and local health informatics systems to inform public health decision-making in HIV transmission cluster investigations. No compensation was provided to participants of the focus groups. 

### 2.3. Study Instrument

A list of semi-structured, open-ended questions was created to enhance discussion in each focus group (Table 1). Questions were chosen to reflect the stakeholder representation at each focus group and to deepen the discussion of specific topics. The questions were emailed to participants ten days prior to each focus group session.

### 2.4. Study Procedures

The focus group sessions were conducted by a primary moderator responsible for leading the discussion and a secondary moderator responsible for notetaking and recording. The primary moderator reviewed the informed consent form with the participants, answered questions, and reaffirmed that participation was voluntary. The focus groups were recorded and transcribed using the professional transcription service Rev (https://www.rev.com/, accessed on 7 October 2019). Identifying information was removed from all written transcripts by one research member to ensure no identifying information was included before other members of the research team accessed the transcription data. One research member listened through all three recordings to confirm the transcribed text.

### 2.5. Data Collection, Collation, and Analysis

Qualitative data were obtained from the focus groups during which the questions, described in Table 1, were posed to the participants and the moderators probed for clarity and depth. Data from all three focus groups were included for analysis. Data were analyzed using a thematic qualitative content analysis framework [16] in which emergent themes were identified and content coding occurred as an iterative process. Two research members independently conducted an initial review of the transcriptions to identify emergent themes related to the potential issues associated with the feasibility, acceptability, interpretation, and utility of phylodynamic-based methods for molecular HIV surveillance. The researchers met weekly to discuss the emergent themes and subthemes, operationalize definitions for each unique thematic code (i.e., node), and resolve discrepancies. A third researcher was consulted for final consensus for any unresolved discrepancies. All coding and qualitative analyses were completed using NVivo version 12 software [17].

## 3. Results

Participants of the focus groups (*n* = 17, 52.9% female) included molecular epidemiologists (4), a computer scientist, infectious disease research scientists (2), and public health professionals (10) at all levels of government (county, state, and federal). Three participants (two public health professionals and one molecular epidemiologist) accepted the invitation to attend more than one focus group. All the sessions were conducted using a hybrid platform in which some participants attended remotely while others attended in person. The accumulated focus group data included 196 min of audio content that was transcribed and coded by emergent theme. Most focus group participants routinely used molecular methods for surveillance. The participants collectively agreed that linking molecular sequence to epidemiological data (from times/locations to demographic/clinical information) is important for improving HIV surveillance. Two major themes, capturing the main issues surrounding the usability and utility of phylodynamic methods for molecular HIV surveillance, emerged from the data: (I) challenges in methodology and (II) implementation concerns. Within each theme, four subthemes arose (Table 2). All eight subthemes were present in every focus group.

### 3.1. Limitations to Current Clustering Methods

**Figure 1:**
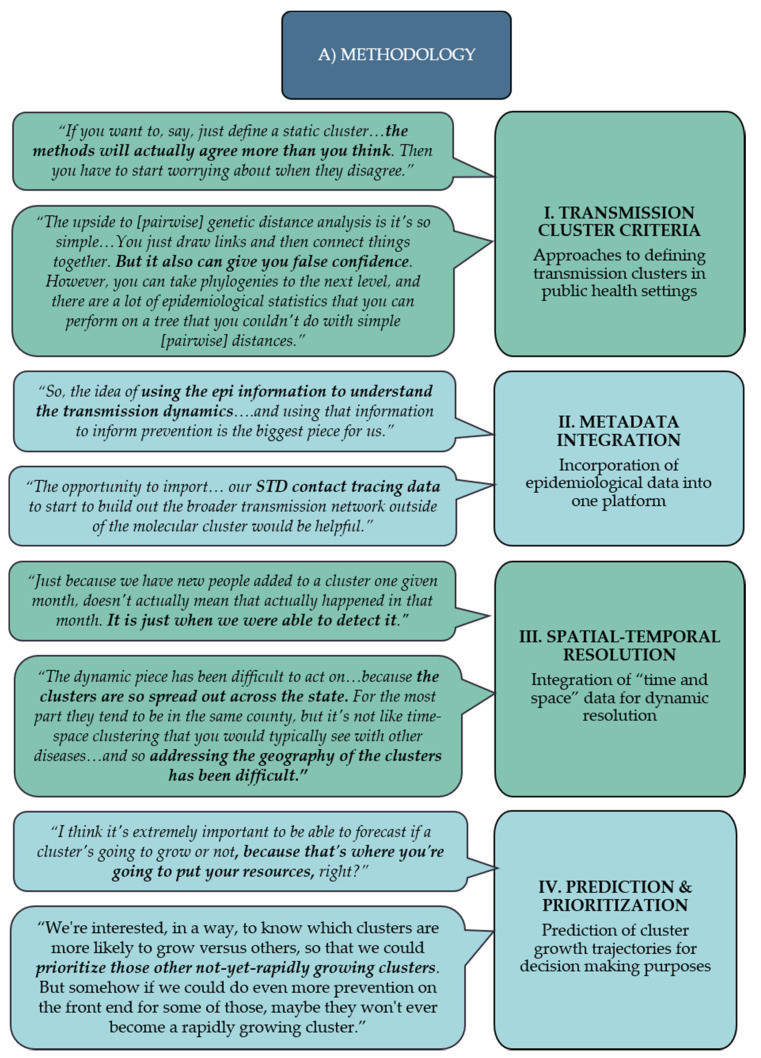
Methodology subthemes and noteworthy quotes from the focus group participants with varying expertise in HIV molecular epidemiology and public health surveillance.

The first major theme to emerge from the data was related to participants’ methodological concerns regarding the use of current and future molecular methods for HIV surveillance. The methodology subthemes included (1) transmission cluster criteria, (2) metadata integration, (3) spatial–temporal resolution, and (4) prediction and prioritization (Figure 1). 

#### 3.1.1. Subtheme I: Transmission Cluster Criteria

Discussion of the different approaches to define HIV transmission clusters in public health settings was classified under subtheme: HIV transmission cluster criteria. This was the most prominent subtheme of the focus group series, accounting for 20.3% (73/360) of all coded references. The conversation concentrated on many of the differences between patristic (i.e., phylogeny-based) and pairwise genetic distance-based methods. Within each genetic distance approach, the different operating definitions for priority transmission clusters, e.g., based on genetic distance thresholds or cluster-linkage methods, were also discussed by the participants. One molecular epidemiology (ME) expert, when comparing the advantages and disadvantages of patristic vs. pairwise distance-based methods, remarked,

“If you want to, say, just define a static cluster…the methods will actually agree more than you think. Then you have to start worrying about when they disagree…The upside to [pairwise] genetic distance analysis is it’s so simple…You just draw links and then connect things together. But it also can give you false confidence. However, you can take phylogenies to the next level, and there are a lot of epidemiological statistics that you can perform on a tree that you couldn’t do with simple [pairwise] distances.”

(FG2, ME expert)

Many participants agreed that phylogenetic methods which use spatiotemporal information (i.e., phylodynamics/phylogeography) can be advantageous over simpler pairwise distance-based methods for tracking the epidemic, and perhaps help with real-time cluster characterization. However, participants were concerned that the expertise needed to perform phylodynamic cluster investigations presents a major obstacle to the uptake of these methods in public health settings, coupled also with the computational burden of estimating phylogenies (especially with Bayesian methods). Within our focus groups, this was the primary reason why pairwise distance-based methods (e.g., HIV-TRACE and MicrobeTrace) were preferred, since many of the bioinformatics steps required, such as sequence alignment, were automated as the platforms did not require multi-sequence alignment programs. Another issue discussed was related to the different definitions of transmission clusters based on genetic distance thresholds, and, the lack of a consensus on the criteria for defining clusters of concern that require heightened monitoring. When asked about how different—and in some cases more restrictive—definitions of transmission clusters affect public health investigations, a public health (PH) official participant remarked,

“I think the limitation is you really put blinders on to the point where you [are] missing the opportunity for intervention.”

(FG3, PH official)

In other words, there is concern that relying on prescribed genetic distance thresholds will increase the potential for intervention opportunities to be missed among clusters not detected through local molecular surveillance.

#### 3.1.2. Subtheme II: Metadata Integration

According to the focus group participants, the current approach to molecular surveillance often involves piecing together fragmented epidemiological data from different sources prior to decision-making. These steps can be labor intensive and time-consuming, ultimately affecting the timeliness with which decisions are made and interventions occur. Discussion related to the incorporation of epidemiological data from multiple sources (for instance, data on genotype sequences, demographics, geography, dates of HIV diagnosis and HIV genotype, transmission risk groups, and viral suppression status) into a unified system was termed metadata integration. When asked about the utility of adding these epidemiological data into molecular surveillance platforms, a public health official emphasized it as an essential component for decision-making,

“So, the idea of using the epi information to understand the transmission dynamics….and using that information to inform prevention is the biggest piece for us.”

(FG2, PH official)

Metadata integration accounted for 43/360 or 11.9% of coded references and was a common topic identified by the participants (particularly the public health officials) when asked how they envisioned a usable and useful software tool for molecular surveillance. The participants collectively expressed a need to have one platform into which they can integrate as much available data as possible, including from sources that remain separate from molecular surveillance (e.g., partner services who report on the risk behaviors and contacts of PLWH). On this topic, a public health official stated,

“The opportunity to import… our STD [sexually transmitted disease] contact tracing data to start to build out the broader transmission network outside of the molecular cluster would be helpful.”

(FG2, PH official)

#### 3.1.3. Subtheme III: Spatial–Temporal Resolution

Dialogue surrounding the integration of “time and space” data, for instance, data on infection recency and geographic location, was termed spatial–temporal resolution. This subtheme represented 7% (25/360) of coded references. Spatial–temporal resolution was identified as an important omission of current clustering methods, representing a major hurdle to using molecular data in real-time investigations. Incorporating spatiotemporal information is key to enable dynamic characterization of clusters. This is true for clustering based on pairwise distances, e.g., reporting the distribution of sampling years of a cluster, as well as for more complex phylodynamic/phylogeographic methods that reconstruct ancestral locations and dates along the branches of a phylogenetic tree.

A research scientist participant remarked,

“I was just imagining a case where in a small town you tested me, and I was positive. And you said, “name your friends,” and I named all of you. And you tested, and we were all positive, but maybe we got infected five years ago. But we just got diagnosed and the reason it looks like a cluster is because well, lo and behold, I just identified you. But that would require different public health. It might alarm you, at that stage, because, wow, it is an emerging cluster. But we have actually been infected for five years then how could you tell that versus something more recently?”

(FG1, research scientist)

A public health official further explained that,

“Just because we have new people added to a cluster one given month, doesn’t actually mean that that actually happened in that month. It is just when we were able to detect it.”

(FG1, PH official)

In other words, current molecular surveillance technologies are not well equipped to distinguish between historical and active clusters, which require different degrees of intervention.

#### 3.1.4. Subtheme IV: Prediction and Prioritization

The subtheme of “prediction and prioritization” was defined as the classification and prediction of cluster growth trajectories, for instance, clusters may be classified as expanding, stabilizing, shrinking, or dividing, to inform public health prioritization schemes. Prediction and prioritization accounted for 38/360 (or 10.6%) of coded references. Currently, no direct methods or software are available to predict and prioritize specific clusters, but conclusions can be drawn through interpretation of networks or phylodynamic trees scaled in time with derived measures [18], such as basic and effective reproductive numbers. Related to this topic, a molecular epidemiology expert emphasized,

“I think it’s extremely important to be able to forecast if a cluster’s going to grow or not, because that’s where you’re going to put your resources, right? You have a big cluster that’s rampantly growing, you’re going to target that cluster, prioritize that cluster ahead of a cluster that’s stable or declining.”

(FG3, ME expert)

A public health official remarked,

“We’re interested, in a way, to know which clusters are more likely to grow versus others, so that we could prioritize those over not-yet-rapidly-growing clusters. But somehow if we could do even more prevention on the front end for some of those, maybe they won’t ever become a rapidly growing cluster.”

(FG2, PH official)

### 3.2. Implementation

**Figure 2:**
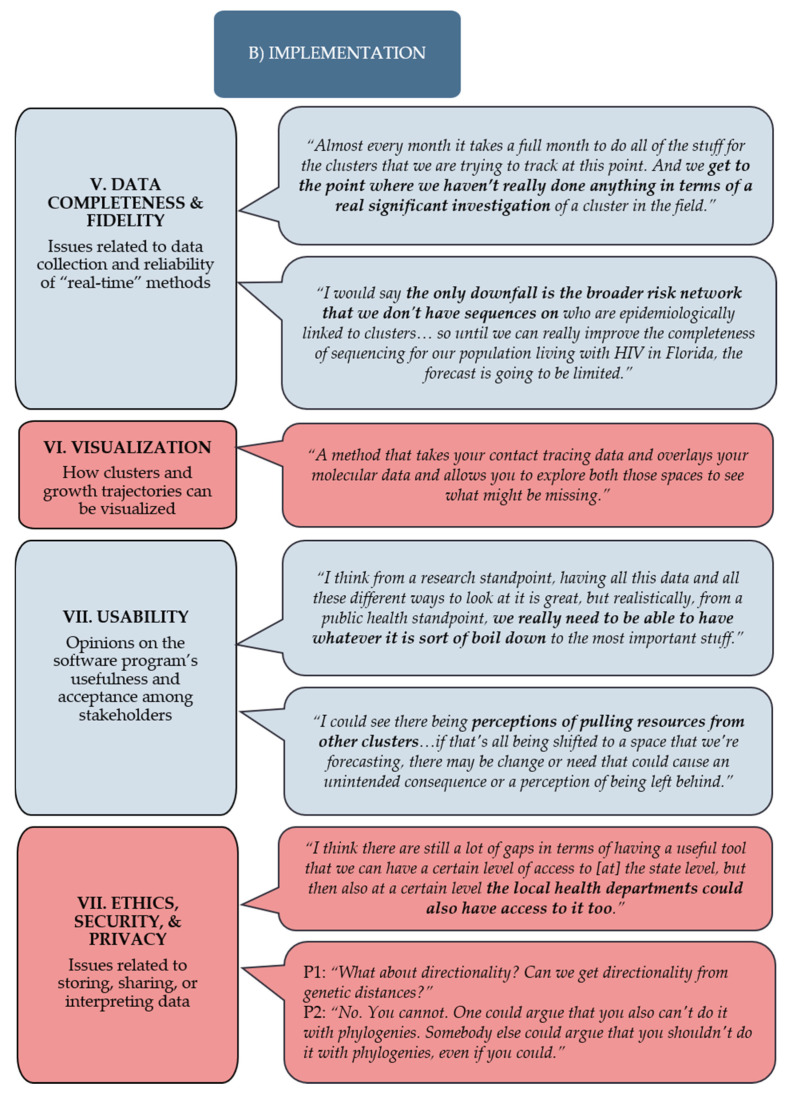
Implementation subthemes and noteworthy quotes from the focus group participants with varying expertise in HIV molecular epidemiology and public health surveillance.

The second major theme to emerge from the qualitative analysis was related to the implementation challenges arising from the incorporation of phylodynamic methods. Emergent subthemes were (5) data completeness and fidelity, (6) visualization, (7) usability, and (8) ethics, security, and privacy (Figure 2).

#### 3.2.1. Subtheme V: Data Completeness and Fidelity

Discussion of issues related to data collection and the reliability of (near-) real-time methods was listed under the implementation subtheme of data completeness and fidelity. This subtheme represented 35/360 (9.7%) of coded references. Participants noted several difficulties with providers being hesitant to order the genotypes needed for public health surveillance. They also commented on issues with receiving sequence data from laboratories in a short enough timeframe to inform real-time prevention efforts. One public health official remarked,

“Almost every month it takes a full month to do all of the stuff for the clusters that we are trying to track at this point. And we have yet to get to the point where we have really done anything in terms of a real significant investigation of a cluster in the field.”

(FG1, PH official)

In addition to time delays, missing sequence data was another issue repeatedly mentioned. A public health official added,

“I would say the only downfall of that is the broader risk network those individuals that we don’t have sequences on who are epidemiologically linked to cluster members could make a huge impact on really what that forecast looks like. And so, until we can really improve the completeness of sequencing for our population living with HIV in Florida, the forecast is going to be limited.”

(FG3, PH official)

#### 3.2.2. Subtheme VI: Visualization 

Dialogue related to the graphical visualization of clusters and corresponding growth trajectories was categorized as “visualization.” This subtheme accounted for 43/360 (11.9%) of coded references. In this section, participants remarked on several desired aspects of an ideal molecular surveillance program, highlighting important visualization limitations of existing programs. A public health official envisioned,

“A method that takes your contact tracing data and overlays your molecular data and allows you to explore both those spaces to see what might be missing, what people you might want to go get a sequence from, just to see if there is actually a linkage between those and maybe even linkages with other STIs [sexually transmitted infections].”

(FG2, ME expert)

#### 3.2.3. Subtheme VII: Usability

Discourse related to participants’ opinions on the software program’s usefulness and acceptance among stakeholders was termed usability. This was the second most prominent theme to emerge from the focus group discussions, accounting for 64/360 (17.8%) of coded references. A public health official reasoned,

“I think from a research standpoint, having all this data and all these different ways to look at it is great, but realistically, from a public health intervention and investigation standpoint, we really need to be able to have whatever it is sort of boil down to: this is the most important stuff to look at, and then, this is how you use it.”

(FG2, PH official)

Related to the anticipation of acceptance among stakeholders, another public health official added,

“I could see there being perceptions of pulling resources from other clusters, like a community response to that, where they also may need resources or support, and if that’s all being shifted to a space that we’re forecasting, there may be change or need that could cause an unintended consequence or a perception of really being left behind in terms of our activities with prevention.”

(FG3, PH official)

#### 3.2.4. Subtheme VIII: Ethics, Security, Privacy

Discussion of issues related to data storing, sharing, and interpretation were categorized under the subtheme: Ethics, Security, and Privacy. This subtheme represented 39/360 (10.8%) of all coded references. Sharing data across multiple jurisdictions was a common issue brought up by the participants who worried that legal restrictions limiting the exchange of data between counties and states that share cluster members would continue to be a major limitation of molecular surveillance. A molecular epidemiology expert remarked,

“I think there are still a lot of gaps in terms of having a useful tool that we can have a certain level of access to [at] the state level, but then also at a certain level, the local health departments could also have access to it too.”

(FG1, ME expert)

Participants also brought up issues related to inferences of transmission directionality (i.e., who infected whom) and whether this information should be included in molecular surveillance even if it were possible.

FG2, ME expert #1: “What about directionality? Can we get directionality from genetic distances?”

FG2, ME expert #2: “No. You cannot. One could argue that you also can’t do it with phylogenies. Somebody else could argue that you shouldn’t do it with phylogenies, even if you could.”

## 4. Discussion

To our knowledge, this was the first study to assess the barriers to implementation of molecular phylodynamic methods for HIV surveillance. This study addressed two major components of HIV molecular epidemiology: (a) transmission cluster identification and (b) phylodynamic cluster forecasting using a human-centered design approach that emphasized end-user input. Participants of the focus groups identified multiple opportunities to enhance current methods and increase software uptake for decision-making purposes in the future. From a molecular epidemiology standpoint, the participants identified several methodological points of discussion. Inconsistent or arbitrary criteria for transmission cluster identification was a common issue voiced by the participants. This finding was consistent with a similar qualitative study in which experts in molecular epidemiology and bioethics expressed a concern about the inconsistent cut-offs used to identify transmission clusters [11]. One way to overcome this issue would be to resolve definitions of transmission clusters between state and federal health departments, considering both local demographics (e.g., rural versus urban classification) and epidemic features (e.g., incidence rates). The recent statistical framework proposed by Chato, Kalish, and Poon (2020) recommends tailoring transmission clustering thresholds to the local epidemic context [19]; however, the current HIV molecular surveillance program infrastructure in the US would likely be unable to facilitate the high level statistical analyses required to accomplish this. Thus, there is benefit to operationalizing threshold definitions for public health settings. Another barrier related to the uptake of molecular methods was the lack of expertise available at the health department level. This was the primary reason why pairwise genetic distance-based methods were preferred since many of the steps required (e.g., sequence alignment and cluster visualization) were automated. To increase uptake of phylodynamic forecasting tools, developers could consider automating as many steps as possible with careful attention to underlying assumptions. Methodologists could also work with public health officials to design training aimed at increasing knowledge levels among staff members. This may become increasingly important in the future, as the use of (deep) machine learning and other artificial intelligence-based algorithms to predict disease spread continue to permeate the HIV molecular surveillance landscape [20,21]. 

From a public health perspective, the participants highlighted several key barriers to implementation. One of the major issues related to use of molecular surveillance in general, is missing data, specifically HIV sequences. To overcome this barrier, public health officials could encourage more providers to order viral genotyping for their patients. Despite being the standard of care, lack of viral genotyping for newly diagnosed PLWH was a prominent issue summarized in subtheme “Data completeness and Fidelity.” Cost of genotyping for health systems with limited budgets has been identified as one potential impediment to increasing HIV genetic sequencing coverage, as well as the declining clinical utility of molecular genotypes for persons initiating first-line integrase strand transferase inhibitor therapies [22]. Further evaluation of the cost-effectiveness of HIV viral genotyping for public health utility is needed. Additionally, health departments should work with commercial laboratories to ensure timely data transfers of molecular sequence data according to state mandates (administrative code 64D3 in Florida). Another issue raised was related to the legalities surrounding data sharing between US states and counties. Currently, jurisdictions with outbreaks spanning beyond their borders are restricted from sharing data or working collaboratively with the other effected regions on molecular surveillance. The participants in our study agreed that increased discourse related to inter-jurisdictional data sharing will be helpful for improving cluster investigations between counties and between states since people and pathogens are mobile, though this approach presents additional privacy concerns. This concept of data sharing and the potential ethical and legal implications surrounding it has been described by molecular HIV surveillance experts and bioethicists in previous qualitative studies [11,13]. Some solutions proposed to overcome these issues include requiring informed consent before using sequence data for public health surveillance and quantifying the minimum depth of sequence data required in a population for effective molecular epidemiology to prevent new HIV infections [13].

This study had limitations. First, the participant sample size was relatively small, which may have resulted in a lack of representation of stakeholders throughout the state of Florida and the US in general. This study also did not have adequate representation of lay persons or PLWH whose perspectives are valuable to understand the barriers facing the implementation of molecular surveillance-based intervention strategies in the community. Moreover, although common in qualitative focus group studies, the method of participant recruitment (purposive, convenience, and snowball sampling) and the study instrument (open-ended questions) are prone to bias and might not be ideal for generalizing conclusions [14]. The hybrid nature of the focus group sessions permitted more participants to attend the discussions, though it may have impacted what participants were willing and able to discuss. Some participants attended more than one focus group and therefore their voices may have had more influence on the results than others. Given that only three focus groups were conducted, and some of which included the repeat participants, it is possible that we may not have had enough data to reach saturation and may have missed other ideas and themes that did not emerge [23]. It is also possible that revealing focus group questions to the participants 10 days in advance could have resulted in bias, as participants could have consulted with their colleagues and employers prior to the focus group sessions; however, these questions were not sensitive in nature and the conversation was often driven by group discussion. Thus, it is unlikely that groupthink bias occurred. Although we did not quantify the level of agreement between coders (e.g., through inter-rater reliability), our method of multiple coding, in which cross-checking of codes occurred iteratively by two independent researchers, was systematic and therefore subjectivity was minimized [24]. Further, since the only state-level public health official participants in the focus groups were from Florida, these findings may not be directly translatable to other states’ epidemics.

## 5. Conclusions

HIV remains a significant source of morbidity and mortality worldwide. In the US, efforts are underway to reduce new infections by 90% over the next ten years. To do this, new molecular phylodynamic-based techniques are under development to dynamically predict infection hotspots before new transmissions occur. To date, however, vital input from the institutional and governmental stakeholders who will deploy these new techniques—at the national, state, and local levels—is lacking. As evidenced from this study, a variety of implementation barriers are expected in relation to the uptake of molecular phylodynamic methods for HIV transmission surveillance in public health settings. Nonetheless, there are several opportunities and promising works-in-progress to enhance current methodology as well as increase the usability and utility of molecular epidemiology software to better guide public health decision-making for HIV prevention.

## Figures and Tables

**Table 1:** Focus group open-ended question/prompt guide.

Focus Group I—Definition of Phylodynamic Methods for the Public Health Official
What is your perception of the utility of linking genetic information to HIV incidence data?
2. Tell us about your use of current clustering programs for HIV.
3. Have you used HIV-Trace, Cluster-Picker, PhyloPart, or other programs? Discuss what you like and dislike about these software programs.
4. What is your perception of the utility of adding temporal resolution to your clustering analyses?
5. How do you envision a software for HIV clustering that is both usable and useful?
Focus Group II—Interrogation of phylodynamic methods by molecular epidemiologists
What is your definition of an HIV transmission cluster and how can it help public health research and interventions?
2. Discuss the advantages and limitations of using a phylogenetic-based approach as opposed to the traditional genetic distance approach for detecting HIV transmission clusters.
3. Have you obtained different results using multiple approaches on the same data?
4. How do you envision phylogenetic transmission clustering to be coupled with epidemiological information to inform public health strategies and interventions?
5. Phylodynamic methods are being developed to include dynamic temporal measures that describe transmission cluster growth, shrinkage, and division events. Do you find this information useful for public health investigations? Are there other measures that you would find useful?
6. How do you envision a software program for the public health official implementing a phylodynamic model? How would you like to feed the data, get results, visualize them, and get informed reports?
Focus Group III—Realistic implementation of phylodynamic methods in public health
What is your perception of the utility of distinguishing between “dynamic” and “static” molecular transmission clusters in public health surveillance?
2. Tell us about your current use of “dynamic” (time and space) data for molecular surveillance. What are the advantages and limitations to using or interpreting these data using current methods (e.g., HIV-TRACE, MicrobeTrace)?
3. What is your perception of the utility of forecasting cluster dynamics? What are the advantages and limitations of using these predictions in public health investigations?
4. How do you envision a software tool that would enable you to view dynamic clusters and forecast their trajectories?

**Table 2:** Subthemes related to the methodological and implementation considerations for the use of phylodynamic methods in HIV molecular surveillance.

Themes	Subthemes	Definitions
Challenges in methodology	Transmission cluster criteria	- Approaches to defining and prioritizing transmission clusters in public health settings (e.g., phylogeny vs. genetic distance-based methods)
	Metadata integration	- Incorporation of epidemiological data (e.g., demographics, geography, transmission risk group, and partner services data) into one platform
	Spatial–temporal resolution	- Integration of “time and space” data for dynamic resolution
	Prediction and prioritization	- Prediction of cluster growth trajectories based on classification (e.g., quantitative evidence of shrinkage, expansion) for decision-making
Implementation concerns	Data completeness and fidelity	- Issues related to data collection and reliability of “real-time” methods
	Visualization	- How clusters and growth trajectories can be visualized in a software program
	Usability	- Opinions on the software program’s usefulness, complexity, and acceptance among stakeholders
	Ethics, security, privacy	- Issues related to storing, sharing, or interpreting data
